# The geography of prescription pharmaceuticals supplied to the USA: levels, trends, and implications

**DOI:** 10.1093/jlb/lsaa085

**Published:** 2021-01-29

**Authors:** Yashna Shivdasani, Neriman Beste Kaygisiz, Ernst R Berndt, Rena M Conti

**Affiliations:** Wellesley College, Wellesley, MA, USA; Wellesley College, Wellesley, MA, USA; Alfred P. Sloan School of Management, Massachusetts Institute of Technology, Cambridge, MA, USA; National Bureau of Economic Research, Cambridge, MA, USA; Boston University Institute for Health System Innovation & Policy, Boston, MA, USA; Boston University Questrom School of Business, Boston, MA, USA

**Keywords:** Food and Drug Administration, prescription drugs, generic, manufacturing, geography, industrial policy, regulation

## Abstract

Prescription pharmaceuticals are frequently used consumer products whose manufacturing location is commonly held as a trade secret by firms and US regulatory agencies. Here we use previously non-publicly available data to describe levels and trends in the manufacturing locations of the most commonly used prescription pharmaceuticals, off-patent generic drugs, intended to be consumed by Americans. We find that the base ingredients required for the manufacturing of these prescription drugs are overwhelmingly and increasingly manufactured in non-domestic locations, specifically India and China. The manufacturing of finished prescription drugs for the American market is more equally split between domestic and foreign locations, but is increasingly foreign as well. The American reliance on non-domestic manufacturing of prescription drugs is important for stakeholders to appreciate, given current quality and pricing concerns and their potential susceptibility to interruptions in supply due to natural disasters, pandemics, and international trade negotiations. We discuss implications of these levels and trends for current domestic and international policy discussions.

## I. INTRODUCTION

This research documents the changing geography of manufacturing for a very commonly used product by American consumers, ‘off-patent’ generic prescription drugs.

Our focus on generic drugs is highly relevant and salient for a number of reasons. The USA is the world’s largest pharmaceutical drug market. Nearly 70 percent of Americans are on at least one prescription drug, and more than half take two.^1^ US demand for generic drugs has grown substantially in the last few decades, reflecting in part the accumulated track record of safety, efficacy, and relatively low prices. More than 90 percent of pharmaceutical drug prescriptions in the USA are currently dispensed as generic drugs.^2^

The geographic sources of generic drugs have also become prominent topics in recent public policy discussions.^3^^,^^4^ While the USA is a world leader in developing and marketing ‘on-patent’ branded prescription drugs, numerous reports have suggested the manufacturing of generic drugs intended for US consumption is increasingly occurring abroad, particularly in China and India.^5^^,^^6^ Vulnerabilities in the supply of drugs, including generics, have been linked to drug quality and safety concerns, and notably geography-based concerns including weather-related events impacting domestic production and controversies involving international trade provisions among foreign manufacturers.^7^ Very high prices and price spikes have also been noted among specific generic prescription drugs in the USA, which may be related to supply or quality concerns.^8^ The recent coronavirus disease 2019 (COVID19) pandemic has heightened concerns regarding American reliance on foreign drugs.^9^

Quality concerns and potential supply interruptions are surprising in part because in order to be marketed in the USA, prescription drugs must meet or exceed minimal but stringent regulatory standards for safety, purity, and efficacy set by the US authority in charge of regulating these products, the US Food and Drug Administration (“FDA”).^10^ Therefore, we begin by summarizing the US market for generic prescription drugs and the role of regulation and policy policies in overseeing product quality and manufacturing geography. The empirical work we present builds on previous analyses that distinguished US from non-US manufacturing sources of generic prescription drugs between 2013 and 2017 based on public data published by FDA.^11^ To place generic drug manufacturing geography levels and trends into broader context, we summarize the existing economic geography literature on why regions and countries specialize in the manufacturing of certain products for domestic consumption and then specialize further by expanding into export markets. This economic literature explains that generic pharmaceutical firms’ manufacturing geography opportunities arise in large part endogenously and reflect historical choices made by firms and public policy makers, including the use of tools such as rules promoting public safety, tax provisions, tariffs, trade policy, and even human capital investments.

## II. INDUSTRY AND PUBLIC POLICIES INCENTIVIZING THE GEOGRAPHY OF PHARMACEUTICAL MANUFACTURING

In this section, we first briefly describe the US market for generic prescription drugs and the role of regulation and policy policies in overseeing product quality and manufacturing geography.

The manufacturing of drugs typically involves several actions, each of which is regulated by authorities in the country where the drug is marketed for final consumption. An initial set of steps involves making essential biochemical ingredients (“raw”, “bulk”, or “starting materials”), and creating “intermediates”. In a second step at the same or different location, these bulk or intermediate materials are combined into a form that is biologically active but not readily consumable by patients (active pharmaceutical ingredients, or “APIs”). ^12^ In the final step, again at the same or yet another location, the API is converted into consumable formulations (final dosage forms, “FDFs”, eg tablets, capsules, ointments, also called “drug products”).^13^

US federal law, as codified by regulations of FDA, oversees the production and sale of prescription drugs in the USA. In the USA, only prescription drugs and their base ingredients that are manufactured in accordance with basic quality manufacturing standards can be legally marketed by manufacturers, whether domestic or internationally based. FDA rules mandate that drugs to be sold in the USA must meet legal requirements for safety, and that they have the quality, purity, identity, and strength that they are represented to possess. These rules are the result of long-standing efforts by the government and private parties to protect the American public from the consumption of potentially harmful substances.^14^^,^^15^

To gain approval to market a new molecular entity in the USA, the sponsor applicant must file a New Drug Application (“NDA”), a dossier containing extensive clinical trial evidence of safety and efficacy and documentation of compliance with Current Good Manufacturing Practice (cGMP) regulations, and obtain approval of the NDA from FDA.^16^ cGMP regulations for drugs contain minimum requirements for the methods, facilities, and controls used in manufacturing, processing, and packing of a drug product.^17^ The regulations ensure that a product is safe for use, and that it has the ingredients and strength it claims to have. The approval process for new and generic drug marketing applications includes a review of the manufacturer’s compliance with the cGMPs. FDA assessors and inspectors determine whether the firm has the necessary facilities, equipment, and ability to manufacture the drug it intends to market.

The requirements to obtain approval to market a generic drug in the USA are considerably less onerous than for an NDA. In particular, the generic sponsor’s Abbreviated New Drug Application (ANDA) need not duplicate the clinical trial evidence regarding safety and efficacy, but instead needs only to provide evidence regarding its pharmaceutical and bioequivalence to the reference drug, as well as manufacturing in conformity with cGMP requirements. This requires the ANDA API or FDF sponsor to submit details regarding the chemistry, manufacturing and controls of a drug component. Components of a drug include the API or drug substance, excipients, and packaging material. When contained in a separate document, such information is called a Drug Master File (“DMF”).^18^ There is no legal or regulatory requirement to file a DMF. Information usually contained in a DMF can instead be provided in an NDA or ANDA.

Generic pharmaceutical companies can manufacture their bulk, API and/or FDF in-house, transfer it to a company affiliate, or outsource it to domestic or ex-US facility contractors. After having obtained approval from the appropriate national health authorities, foreign generic companies can export API or FDF to a US affiliate company, contract to sell API/FDF to generic companies in other countries, or can use their API or FDF for domestic sales of their own drug product. Traditionally, European-based API companies have focused on selling to branded companies in the USA and Western European markets because of the Europeans’ proven capabilities to manufacture products meeting high quality standards.^19^

However, the industry trade press has noted that a changing trend is that far East Asian API companies are becoming major suppliers to the generic industry manufacturing drugs intended for consumption in Western markets.^20^ Based in part on the concern that foreign manufacturing sites were not being inspected as frequently and as thoroughly as US API and FDF manufacturing sites, in 2012 Congress passed the Generic Drug User Fee Act (GDUFA) legislation, authorizing FDA to collect application and annual fees from ANDA applicants and ANDA holders. FDA committed that it would use some of the GDUFA user fee revenues to inspect foreign sites on a similar schedule to that of domestic sites.^21^

Based on data recently made public by FDA, our prior research documented that between 2013 and 2017, the vast majority (almost 90 percent) of sites manufacturing API for pharmaceutical products intended for domestic US consumption were located outside the USA, and that a smaller majority (about 60 percent) of FDF manufacturing sites were foreign, with both API and FDF facilities becoming increasingly foreign over time.^22^^,^^23^ By 2017, the number of domestic FDF facilities was about two and one-half times larger than the number of domestic API facilities. However, between 2013 and 2017, the total number of manufacturing facilities (domestic plus foreign) registered to supply API and FDF to the US market fell, with the USA shedding about 21–22 percent of FDF and API facilities, foreign suppliers reducing their number of API facilities approximately half that much (about 10 percent) and cutting their number of FDF facilities by a much smaller proportion, about 3 percent.

These results imply the domestic manufacturing of off-patent prescription drugs includes primarily FDF rather than API activities, and that the growing vast majority of API generic drug manufacturing intended for US consumption is now occurring abroad.^24^

### II.A. The Economic Geography of Pharmaceutical Manufacturing

#### II.A.1. An overview of historical factors affecting the location of generic drug manufacturing

In this section, we document that he current geography of generic drug manufacturing for drugs intended for US consumption may reflect in large part prior industry and public policies that facilitated exploitation of scale and agglomeration economies, along with particular labor force skills.

Solid oral drugs are light in weight, are small in volume, and in most cases are robust to being shipped in large containers such as tank rail cars or air or shipping freight containers. Relative to their commercial value, transportation costs for drugs are modest at most, and since most drugs today are synthetic and not derived from soil samples or botanical plants, the location of API or FDF manufacturing does not depend critically on natural resource endowments. Hence, the geographic location of API and FDF pharmaceutical manufacturing is unlikely to be substantially explained by the Hecksher–Ohlin theory of comparative advantage based on natural resource endowments—a theory that posits that trade between regions is based in large part on endowment differences among regions, with regions tending to produce relatively more of and export goods that are intensive in the resource factors with which they are abundantly endowed.^25^

An alternative set of theories explaining why countries and regions specialize in production and trade involves the exploitation of economies of scale and agglomeration economies. While the notion of scale economies is quite straightforward (on which we elaborate below), some authors have distinguished internal from external scale economies^26^, while others interpret external economies of scale as an example of agglomeration economies.

External economies of scale occur when the cost per unit of output depends on the overall size of the industry, but not necessarily on the size of any one firm. An industry where economies of scale are purely external will typically consist of many small firms and often be very competitive, but there will be no intrinsic advantage to larger firms.

The analysis of external economies was originated by the British economist Alfred Marshall who was struck by the phenomenon of “industrial districts” or clusters—geographical industry concentrations that could not be explained by natural resources.^27^ The notion of external scale economies overlaps substantially with the concept of agglomeration economies:

“Agglomeration economies are the benefits that come when firms and people locate near one another together in cities and industrial clusters. These benefits all ultimately come from transport savings: the only real difference between a nearby firm and one across the continent is that it is easier to connect with a neighbor. Of course, transportation costs must be interpreted broadly, and they include the difficulties in exchanging goods, people, and ideas.”^28^

Hereafter, in describing factors contributing to the choice of manufacturing location, we will not distinguish between external scale economies and agglomeration economies, and refer to the combined effects of internal and external scale economies and agglomeration economies as reflecting economies of scale and agglomeration.

Economies of scale derive in large part from specialization and the division of labor. A particularly important source of scale economies in the manufacturing of API pharmaceuticals is the prominent role of vats, barrels, tanks, and stainless steel reactors—containers holding solids and liquids used in the later-stage manufacturing processes of chemically synthesized small molecules. Economies of scale at the product- and plant-specific level can be attained by expanding the physical size and number of API individual processing units.^29^ Moreover, since the crew needed to operate a large processing unit or machine is often little or no larger than what is required for a unit of smaller capacity, labor costs per unit fall sharply with scale-up. These relationships can operate at the API product, plant and multiplant level and thereby underpin API scale economies at various levels of aggregation.

Regarding agglomeration economies, Marshall argued there are three related reasons why a geographic concentration of agglomerated firms may be more efficient than an individual isolated firm: (i) the ability of a cluster of firms to support a sufficiently viable and sustainable market for critical specialized equipment suppliers, such as for the most complex FDF operations; (ii) a geographically concentrated industry allows labor pooling for workers with highly specialized skills, providing them a source of reliable employment, such as manufacturing process and regulatory compliance consultants for FDF manufacturing. Both workers and employers are better off if all are near each other; and (iii) a geographically concentrated industry helps foster informal knowledge spillovers that often are most prevalent when an industry is concentrated in a fairly small area, so that employees of different companies mix socially and talk freely about technical and regulatory compliance issues. Note that with each of these three sources of agglomeration economies, as industry size increases, the costs of available inputs decline, providing the agglomerated firm clusters with competitive cost advantages.

But this raises a more fundamental issue: from where did these agglomeration economies originally emanate? The answer is usually historical accident; something gives a specific location an advantage in a particular industry, and this advantage gets “locked in” by agglomeration economies even after the circumstances that created the initial advantage are no longer relevant. ^30^ For example, that many FDF manufacturers located their US sites in the mid-Atlantic and Other East states so that they were located not only near the eastern population centers and commercial headquarters, but also were within easy travel distance to the FDA regulators, is unlikely a coincidence. Nor is it likely a coincidence that because of low transport costs and possible exploitation of scale economies far from population centers, that API facilities in the USA have been more geographically dispersed than FDF facilities.

More generally, since manufacturing operations are highly regulated by the FDA and the manufacturing of FDF products is more complex and is likely more susceptible to adulteration than that for low transport cost commodity-like APIs, it is plausible that local regulatory oversight advantages exist for FDF manufacturers that make it preferable to locate their FDF manufacturing sites closer to the location of final consumption than their API sites. While this preference for FDF manufacturing to be close to the location of final drug consumption may be important within the USA, it is likely to be even a more prominent issue for the decision to locate an FDF plan in the US or ex-US.

While scale economies may have a significant impact on the siting of API manufacturing facilities, the possibilities for exploiting economies of scope may apply similarly for API and FDF manufacturing. When there are shared manufacturing inputs, such as boilers, pipes, and stainless steel reactors, manufacturing multiple products at a given plant can yield cost savings relative to manufacturing the various products in stand-alone plants; these cost savings are called *economies of scope,* and derive both from specialized equipment (eg machines making and filling syringes for various injectable drugs, making various oral solid drugs with tablet presses, providing sterile and aseptic storage areas for temporary inventory of goods in process) or from specialized labor (equipment operators, sterile manufacturing supervisors)*.* These scope economies could be present both for API and FDF manufacturers. Scope economies also occur frequently outside manufacturing in other functions integral to the firm that may be particularly important for FDF plants. For example, a company’s regulatory affairs specialist can liaise with a single regulatory body regarding a number of FDF products simultaneously. Sales and marketing personnel can promote numerous FDF products simultaneously.

These considerations raise the issue of how scale, scope, and agglomeration economies affect trends in the location of generic drug manufacturing. The economic literature notes that firms face incentives to invest in capacity expansion when there are scale economies, for such investment will reduce unit costs and enable them to price competitively, assuming they can sell their expanded capacity output. Firms may also pursue rapid market share at lower current profits when they expect to realize learning curve benefits in the future. Regarding both scale and agglomeration economies, public authorities face pressures to protect domestic industries currently experiencing competitive challenges, subsidizing “infant industry” firms with favorable tax treatment and/or protecting them with tariffs, adopting import substitution policies in the hope that as a result imports will be reduced, exports will be increased, scale effects promoted, and prospects for domestic employment and a viable domestic manufacturing industry will be enhanced.^31^

Similarly, recognizing that because of historical accident certain geographical regions have nascent clusters of firms that are manufacturing biopharmaceutical products, public authorities may seek to create environments conducive to generating agglomeration economies, such as providing job skill training and other educational opportunities, establishing tax-free manufacturing zones to attract further biopharmaceutical investments, and incentivizing complementary industries to locate nearby. In the specific context of the pharmaceutical industry, this implies the co-location of chemical, boiler and vat equipment suppliers, providers of inventory storage facilities, and process engineering consulting firms.

The onus on pharmaceutical companies to ensure the quality of prescription drugs intended for sale in the US market likely places additional incentives on firms to identify scale and agglomeration economies of related to satisfying regulatory requirements. The costs of overseeing these firms’ quality production might also guide policymakers to prefer the sourcing of these products from some locations over others. To the extent selling costs to other geographic jurisdictions are affected by import restrictions, tariffs, fees, and regulatory submission requirements, the increased selling, distribution, and transportation costs may offset declines in unit production costs from scale economies, agglomeration effects, or from learning curves, and thereby constrain interregional trade.

From this perspective, we posit that the location of API manufacturing facilities is heavily dependent on scale economies and low transport costs, whereas the location of FDF facilities is dependent on the combination of commercial and regulatory requirements existing in the country or region where the final consumption of the drug product occurs. As a result, we expect that API manufacturing is more geographically dispersed globally than is FDF manufacturing, and that FDF manufacturing sites are more likely in the country or region where the final consumption of the drug product occurs. Moreover, from the perspective of economic geography, the larger proportionate reduction in API than FDF facilities, both domestic and foreign, may be the result of firms’ attempting to capture scale economies by increasing plant capacity, and for US firms, exploiting comparative advantages by outsourcing generic drug manufacturing to foreign FDF, and especially API suppliers.

### II.B. Implications of Scale and Scope Economies for Industry Strategy and Public Policy

The existence of and prospects for realization of scale, scope, and agglomeration economies has important implications for industry strategy, as well as for public policy toward industry. Specifically, in the market for generic drugs historical industry and public policy developments in four regions have had a major impact on the current sourcing of drugs destined for US consumption. In this section, we briefly describe how current manufacturing activities in Puerto Rico, India, China, and Ireland reflect previous industry and public policies.

#### II.B.1 Puerto Rico

For most of the late twentieth century, Puerto Rico was one of the major sources for the mainland US pharmaceutical market, but since then Puerto Rico’s role as a pharmaceutical supplier to the USA has diminished.^32^ Puerto Rico’s role in the US pharmaceutical market can be directly traced to targeted policies fostering scale and agglomeration economies. In 1976, the US Congress created special tax provisions to incentivize firms to locate manufacturing plants and bring medium to high-skilled jobs to Puerto Rico and other US possessions. Also known as the Possession Tax Credit, Section 936, this legislation incentivized the location of pharmaceutical manufacturing in Puerto Rico by granting USA-based corporations a tax exemption on income earned in Puerto Rico.^33^ Subsidiaries of branded and generic companies could develop a drug in their US research and development facilities, transfer the patent or proprietary technical knowledge to their wholly owned subsidiaries operating in Puerto Rico, and claim the income from the drugs as tax-free income. Many branded pharmaceutical, biotechnology, and medical device companies opened manufacturing plants in Puerto Rico. Generic pharmaceutical companies such as Teva, Ivax, and Watson also located manufacturing plants in Puerto Rico following passage of the Section 936 legislation.^34^

Congress voted to phase out Section 936 in 1996, citing excessive cost and the very limited number of US companies that received the tax break. In 2006, the phase-out was completed, resulting in plant closures and declining employment, and imposing an allegedly disproportionate tax burden on domestic Puerto Rican companies. ^35^ Subsequently, the island’s economy was damaged by passage of the US Tax Cuts and Job Act of 2017, since it targeted income from “intangible” assets such as pharmaceuticals and medical devices, on which Puerto Rico has been heavily reliant. ^36^ The Puerto Rican economy was dealt a further blow in 2017 when the island was hit by the devastating Hurricane Maria.^37^

#### II.B.2 India

After gaining Independence in 1947, India’s government pursued the goals of attaining national self-sufficiency in pharmaceutical manufacturing and simultaneously providing its large population with access to low-priced medicines. Specifically, India replaced product patent protection with process patent protection. ^38^ A byproduct of this national import substitution policy was that India’s scientific and technology-trained labor force learned how to reverse engineer imported patented medicines, a skill that became a unique comparative advantage. Moreover, national tax and subsidy policies favored the co-location of manufacturing sites through the establishment of Special Enterprise zones.^39^^,^^40^ In an attempt to exploit further potential scale and scope economies by expanding its export of pharmaceuticals, in 1995 India applied for membership in the World Trade Organization. To do so, in 2005 India needed to comply with the Agreement on Trade-Related Aspects of Intellectual Property Rights, which in turn required India to reintroduce intellectual property protection for product patents, and recognize both process and product patents for 20 years after issue, with some qualifications.^41^

In the years that followed, India’s became a major exporter of APIs, through consolidation and capturing of agglomeration economies, and shifted from API to FDF manufacturing. Price controls were introduced, and fierce competition between thousands of small- and medium-sized firms resulted in a decline in the number of small, inefficient firms that either exited the market or were acquired by larger Indian or foreign firms seeking to take advantage of scale economies, thereby becoming competitive with large multinational companies. Many Indian companies repurposed themselves as contract manufacturers, producing bulk and intermediate products, APIs for new chemical entities, or APIs or FDFs for generic drugs in cooperation with foreign multinational companies. In 2016, about 35 percent of the APIs manufactured in India were exported to the USA, UK, or Japan. In turn, approximately 32 percent of domestic consumption of APIs were imported, with China alone accounting for 57–60 percent of the APIs by rupees imported by India.^42^

In recent years, India’s export-oriented manufacturers have become more selective in launching FDF products abroad, focusing more on high margin specialized pharmaceuticals and biologics, and less on high volume but low margin small molecule oral solid medicines, be they API or FDF products.^43^ As one analyst has observed:

“Starting from around late 2018 to early 2019, traditional generics bigwigs Teva, Mylan, Novartis’ Sandoz, Amneal and Endo have lost out to a group of six competitors that include Indian drugmakers Aurobindo Pharma, Lupin, Dr. Reddy’s, Sun Pharma, Cipla and Canada’s Apotex in terms of weekly total prescriptions … … Amid increased pricing pressure and competition in the generic arena, higher-margin complex generics and biosimilars have lately been put at the top of the growth agenda at Teva, Mylan and Sandoz, the top three US generics players by total prescriptions in 2017. The old idea of ‘first in, last out’ or just waiting out lower-priced competitors until they give up and exit has died … Companies are no longer trying to drive as much volume as possible, but rather are focused on the margin of those sales … ‘Old guard’ firms are filing as many US generic applications as before, but they’re being more careful about which ones they launch … .Companies these days don’t always choose to launch generics even though they are receiving approvals at a similar or greater pace.”^44^

#### II.B.3. China

As a legacy of China’s pre-reform command economy, in the 1970s and 1980s numerous state-owned enterprises produced tablet dosage forms and distributed them to hospitals, their primary consumer. Governments or rural collectives took control of all funds, spent them, but had little incentive to monitor manufactured pharmaceuticals for safety and quality. Potential scale and agglomeration economies were not pursued nor achieved.^45^

Most Chinese manufacturers were not capable of supplying pharmaceuticals to Western regulated markets, choosing instead to focus on the immense local patient populations. Many manufacturers relied on the repetitive production of low value-added bulk pharmaceuticals and imitation drugs and struggled to survive.^46^ Also plaguing Chinese manufacturing of pharmaceutical products were major issues in intellectual property rights protection enforcement. This has resulted in a low level of market protection for domestic branded drugs and allowed established foreign generics and off-patent brands to dominate the domestic market.^47^ Moreover, although China officially has sought to move up the value chain from manufacturing bulk chemicals and intermediate products to producing and exporting finished drugs, the relatively hefty facility and application fees associated with the 2013 implementation of the USA’ GDUFA may be sufficiently prohibitive to limit the number of Chinese companies focusing on pursuing the finished drug US market, instead preferring to invest in the rapidly growing and less regulated domestic Chinese market.^48^^,^^49^

Attracted by China’s comparative advantages in lower costs of manufacturing intermediate chemical goods, lower investment costs, shorter lead times and access to the immense Asian talent pool, in the last decade foreign pharmaceutical manufacturers have invested in the Chinese pharmaceutical industry. These investments were initially limited to the outsourcing of old products and commodity base ingredients involving less sophisticated chemistry.^50^ The Chinese bulk drugs market has evolved rapidly over the years, and today it is large and diversified with about 7000 base ingredient manufacturers.^51^

#### II.B.4. Ireland

Finally, Ireland’s current manufacturing capacity reflects policies similar in some respects to Puerto Rico in previous eras but differ from those in India and China. In Ireland, public policies have primarily used corporate tax provisions to incentivize foreign firms to establish their nominal headquarters in Ireland, even if the multinational firms’ *de facto* operations are managed abroad. Although the tax provisions target corporate headquarter locations, they are linked to the manufacturing of products with intellectual property protection, such as branded drugs.^52^^,^^53^ Outside Ireland, these tax policies are said to incentivize tax inversions.

There are many generic drug manufacturers supplying the American market that have either succeeded or at least attempted to engage in corporate tax inversions, including Endo, Pfizer, Mallinckrodt, Perrigo, Alkermes, Shire and Horizon. ^54^ USA-controlled firms represent almost all foreign firms in Ireland. Academic research has ranked Ireland as the world’s largest tax haven, even larger than the entire Caribbean tax haven system.^55^

However, beginning in 2017 and continuing thereafter, US and UK tax policy countermeasures to stem the flow of Irish tax inversions appear to have had the effect of mitigating the locating of drug manufacturing in Ireland. According to one source, since 2008 the pharmaceutical industry has invested close to €10 billion in manufacturing and research and development in Ireland.^56^

To summarize, in each of these four regions, historical industry strategy and public policy developments facilitating exploitation of scale and agglomeration economies have created an environment resulting in the regions currently playing a prominent role in the manufacturing of generic drugs intended for consumption in the USA. Some of these policies have already been terminated, and many appear malleable to changes in future alterations. Taken together, however, these policies have resulted in the USA being heavily dependent on foreign supplies of API and FDF products.

## III. DATA FOR EMPIRICAL ANALYSES AND METHODS

With these public policies and industry strategies as background, we now move on to a discussion of our new research findings, beginning with a discussion of data sources.

### III.A. FDA Data Underlying Previously Published Research

The data underlying our previous research on the changing US and ex-US shares of API and FDF manufacturing sites for generic drugs intended for US consumption were based on information industry voluntarily supplied to FDA. The data were drawn from two distinct FDA data gathering initiatives.^57^ First, as part of its budgeting process in anticipation of implementing the 2013 Generic Drug User Fee (GDUFA-I) program and setting annual facility site fees, FDA requested information from all firms manufacturing pharmaceuticals regarding the location of their API and FDF sites, domestic and foreign. Then, when announcing its GDUFA-I annual fee assessments in the *Federal Register* 60 days prior to the beginning of each fiscal year (with differential annual assessments for foreign and domestic sites, and for API and FDF facilities), FDA published aggregate counts of US and ex-US API and FDF sites, but did not publish identities or addresses of the sites.

Second, because the revised fee structure in the renewed 2017 GDUFA-II legislation entailed assessment of differential annual fees depending on the number of ANDAs owned by each ANDA holder, FDA needed to determine the current ownership of ANDAs. This was not a trivial data procurement task. While FDA had information on the identity of the original applicant on each currently approved ANDA, there was a consensus at FDA that many approved ANDAs were no longer being marketed. In addition, the current ANDA owners may not be the same as those recorded on the initial ANDA approval or on subsequent communications between the industry ANDA sponsor and FDA, given consolidation among ANDA holders over the years. Thus, in a series of *Federal Register Notice* requests to industry issued in 2016 and 2017, FDA asked ANDA holders to claim all ANDAs owned by them or their affiliates, and to correct any errors on earlier draft spreadsheets distributed by FDA in March 2017, and updated in May 2017. Although the FDA-distributed lists identified the names of the ANDA holders and the approval numbers of their ANDA portfolios, no information was provided regarding where the drugs were currently being manufactured.

In Berndt, Conti and Murphy [2018], we presented tables on the aggregate number of API and FDF sites, US and ex-US, annually 2013–2017 (from the first data source), and on the ANDA size distribution and ANDA portfolio ownership distribution as of April 30, 2017 (from the second data source).

### III.B. New FDA Data Sources

The new research we report here is based on previously nonpublic data made available to us by FDA, drawn from a list of firms participating in and paying annual user fees to FDA’s Generic Drug User Fee program.^58^

The new data extend our previous research in three directions. First, although our earlier research comprised the first five fiscal years of the initial Generic Drug User Fee Program (denoted GDUFA-I) encompassing fiscal years 2013–2017, the new data add the first two years of the renewed user fee program (denoted GDUFA-II)—2018 and 2019—and also provides an alternative count of aggregate US and ex-US API and FDF sites during the GDUFA-I era, enabling a comparison and check on our previous findings. FDA officials have informed us that the count and identity of manufacturing sites in the two data sources (one voluntary—discussed above, the other drawn from mandated user fee payments) do not always agree. Since the latter GDUFA list is constructed based on an accompanying actual payment by the organization remitting the mandated GDUFA fees to FDA, we consider this second source of data more reliable than the data based on the voluntary self-identification data used in previous research.

Second, in our prior research we only had access to counts of API and FDF manufacturing sites geographically aggregated to total US and total ex-US levels, but had no additional information on their address and geographically disaggregated addresses. The new data list the name of the organization remitting the GDUFA fees for each fiscal year, and the detailed address of the manufacturing site (but not necessarily the address of the sponsor organization owning the ANDA and remitting the user fee). Hence, in the current research we combine data from various countries in FDA data base into three global regions: (i) the Americas—USA, Canada, Mexico, Argentina, and Other Americas; (ii) Europe—France, Germany, Italy, Great Britain, Ireland, Rest of Western Europe, and Rest of Eastern Europe; and (iii) Asia and Rest of World—India, China, Israel, South Korea, Taiwan, and Rest of World. For each of these three global regions, we also provide site counts annually for each of the component countries or regions.

For the USA, we also utilize FDA’s definition of its 19 districts, with the component states/regions.^59^ FDA defines its 19 regions as follows: ATL (Georgia, North Carolina, South Carolina); BLT (Maryland, District of Columbia); CHI (Illinois); CIN (Kentucky, Ohio); DAL (Arkansas, Oklahoma, Texas); DEN (Colorado, New Mexico, Utah, Wyoming); DET (Indiana, Michigan); FLA (Florida); KAN (Iowa, Kansas, Missouri, Nebraska); LOS (Arizona, Southern California); MIN (Minnesota, North Dakota, South Dakota, Wisconsin); NWE (Connecticut, Maine, Massachusetts, New Hampshire, Rhode Island, Vermont); NWJ (New Jersey); NOL (Alabama, Louisiana, Mississippi, Tennessee); NYK (New York); PHI (Delaware, Pennsylvania); SAN (Northern California, Hawaii, Nevada); SJN Puerto Rico, US Virgin Islands); and SEA (Alaska, Idaho, Montana, Oregon, Washington). We also combine the 19 FDA districts into four aggregated domestic geographic zones: (i) *Southeast* (ATL, FLA, NOL, SJN); (ii) *Other East* (BLT, NWE, NWJ, NYK, PHI); (iii) *Central* (CHI, CIN, DAL, DET, KAN, MIN); and (iv) *West* (DEN, LOS, SAN, SEA).

Third, although the GDUFA program assesses differential annual user fees for API and FDF manufacturing sites, a well-known phenomenon is that a manufacturing facility site is not a dedicated API site nor a dedicated FDF site, but instead occasionally combines both API and FDF manufacturing at a single site. The data source used in our prior research simply provides counts of total API and FDF sites and does not acknowledge the existence of combined sites. Therefore, that data cannot tell us how many of the API sites are dedicated and how many are combined API and FDF sites, thereby potentially rendering unreliable and ambiguous the count of number of API and number of FDF sites. Based on information provided us by FDA,^60^ we have learned that under GDUFA-I, a facility that qualified as both API and FDF was required to pay both API and FDF annual fees. Under GDUFA-II, however, that facility is only required to pay the FDF fee, which is larger than the API assessment. The information provided by FDA enabled us based on lists of firms participating annually in the GDUFA programs, to distinguish and separately count dedicated API sites, dedicated FDF sites, and combined sites, disaggregated globally and by US FDA district, 2013–2019.^61^

Some limitations remain. While the new FDA data provide more information than did the initial FDA data, neither the initial nor the new FDA data sets disclose what molecules and what formulations (eg tablet, capsule, injectable, infusible, other) are manufactured at the dedicated API, dedicated FDF, or combined sites, any information on corporate ownership relationships of or among the sites, or any information regarding the volumes or dollar values of prescription pharmaceuticals produced at the sites. Moreover, FDA data only incorporate information on generic and prescription pharmaceuticals and do not include information on branded or over-the-counter manufacturing activities.

## IV. RESULTS

### IV.A. Site Counts by Year and US and EX-US Geographic Locations

In the bottom rows of Table 1 we report the number of API (the set of columns in the left panel), FDF (columns in the middle panel), and combined API and FDF sites (columns in the right panel), by fiscal years 2013–2019, aggregated to the US and ex-US geographic totals. The bottom rows of Table 2 report corresponding shares of global totals.

**Table 1:** Number of dedicated API, dedicated FDF, and simultaneous API and FDF sites by global region, FY 2013–2019

	**Number of dedicated API sites**							**Number of dedicated FDF sites**							**Number of simultaneous API and FDF sites**						
**Global regions**	**2013**	**2014**	**2015**	**2016**	**2017**	**2018**	**2019**	**2013**	**2014**	**2015**	**2016**	**2017**	**2018**	**2019**	**2013**	**2014**	**2015**	**2016**	**2017**	**2018**	**2019**
**Americas**																					
USA	114	125	113	110	106	110	103	259	266	267	266	274	288	259	46	29	29	25	24	27	44
CAN	13	14	14	16	14	15	12	29	30	30	30	30	30	22	7	2	2	2	2	2	2
MEX	9	12	10	11	13	12	9	4	3	3	4	4	4	3	2	0	0	0	0	0	1
ARG	4	5	4	3	4	5	4	3	2	3	3	3	2	2	1	1	1	1	1	1	1
Other	4	5	4	4	5	5	4	3	3	4	2	2	2	2	2	1	1	2	1	1	1
**Sub-total**	**144**	**161**	**145**	**144**	**142**	**147**	**132**	**298**	**304**	**307**	**305**	**313**	**326**	**288**	**58**	**33**	**33**	**30**	**28**	**31**	**49**
**Europe**																					
FRA	29	29	27	24	22	25	20	8	12	12	13	11	9	9	1	1	1	4	3	3	6
GER	33	37	35	36	34	33	31	23	24	29	25	26	21	23	6	7	5	5	4	3	2
ITA	66	65	66	64	64	67	61	20	22	19	19	20	20	21	9	8	5	5	5	5	6
GBR	13	14	15	14	13	13	14	5	8	6	8	7	6	5	6	4	1	2	1	2	1
IRL	5	6	7	7	7	5	6	5	5	7	5	4	4	5	2	0	0	0	0	0	1
Rest – Eastern	28	29	30	29	30	27	27	18	20	16	14	11	13	15	6	8	9	9	6	5	5
Rest – Western	87	91	88	86	83	87	84	33	36	40	42	46	47	45	15	9	9	7	9	10	13
**Sub-total**	**261**	**271**	**268**	**260**	**253**	**257**	**243**	**112**	**127**	**129**	**126**	**125**	**120**	**123**	**45**	**37**	**30**	**32**	**28**	**28**	**34**
**Asia Rest—World**																					
IND	208	227	219	215	220	231	222	118	126	138	135	142	143	147	42	26	31	30	32	30	43
CHN	148	160	161	165	160	149	134	28	35	35	43	46	47	49	19	15	17	17	14	17	24
ISR	6	8	8	8	8	7	7	6	5	6	6	6	7	6	2	1	1	1	1	1	1
TWN	17	16	15	15	14	14	14	10	12	14	13	15	17	16	3	4	4	2	1	2	4
SKR	7	6	7	8	7	8	7	0	0	0	1	0	0	1	2	1	0	1	0	0	0
Rest—World	38	39	41	39	36	38	36	16	19	20	16	16	16	16	9	9	10	3	4	5	5
**Sub-total**	**424**	**456**	**451**	**450**	**445**	**447**	**420**	**178**	**197**	**213**	**214**	**225**	**230**	**235**	**77**	**56**	**63**	**54**	**52**	**55**	**77**
**Total US by type**	**114**	**125**	**113**	**110**	**106**	**110**	**103**	**259**	**266**	**267**	**266**	**274**	**288**	**259**	**46**	**29**	**29**	**25**	**24**	**27**	**44**
**Total ex-US by type**	**715**	**763**	**751**	**744**	**734**	**741**	**692**	**329**	**362**	**382**	**379**	**389**	**388**	**387**	**134**	**97**	**97**	**91**	**84**	**87**	**116**
**Total Global by type**	**829**	**888**	**864**	**854**	**840**	**851**	**795**	**588**	**628**	**649**	**645**	**663**	**676**	**646**	**180**	**126**	**126**	**116**	**108**	**114**	**160**
**Total global sites**																					
**Over all types**	**1597**	**1642**	**1639**	**1615**	**1611**	**1641**	**1601**	**1597**	**1642**	**1639**	**1615**	**1611**	**1641**	**1601**	**1,597**	**1,642**	**1,639**	**1,615**	**1,611**	**1,641**	**1,601**

**Table 2:** Share of dedicated API, dedicated FDF, and simultaneous API and FDF sites by global region, FY 2013–2019

	**Share of dedicated API sites**							**Share of dedicated FDF sites**							**Share of simultaneous API and FDF sites**						
**Global regions**	**2013**	**2014**	**2015**	**2016**	**2017**	**2018**	**2019**	**2013**	**2014**	**2015**	**2016**	**2017**	**2018**	**2019**	**2013**	**2014**	**2015**	**2016**	**2017**	**2018**	**2019**
**Americas**																					
USA	13.75	14.08	13.08	12.88	12.62	12.93	12.96	44.05	42.36	41.14	41.24	41.33	42.60	40.09	25.56	23.02	23.02	21.55	22.22	23.68	27.50
CAN	1.57	1.58	1.62	1.87	1.67	1.76	1.51	4.93	4.78	4.62	4.65	4.52	4.44	3.41	3.89	1.59	1.59	1.72	1.85	1.75	1.25
MEX	1.09	1.35	1.16	1.29	1.55	1.41	1.13	0.68	0.48	0.46	0.62	0.60	0.59	0.46	1.11	0.00	0.00	0.00	0.00	0.00	0.63
ARG	0.48	0.56	0.46	0.35	0.48	0.59	0.50	0.51	0.32	0.46	0.47	0.45	0.30	0.31	0.56	0.79	0.79	0.86	0.93	0.88	0.63
Other	0.48	0.56	0.46	0.47	0.60	0.59	0.50	0.51	0.48	0.62	0.31	0.30	0.30	0.31	1.11	0.79	0.79	1.72	0.93	0.88	0.63
**Sub-total**	**17.37**	**18.13**	**16.78**	**16.86**	**16.90**	**17.27**	**16.60**	**50.68**	**48.41**	**47.30**	**47.29**	**47.21**	**48.22**	**44.58**	**32.22**	**26.19**	**26.19**	**25.86**	**25.93**	**27.19**	**30.63**
**Europe**																					
FRA	3.50	3.27	3.13	2.81	2.62	2.94	2.52	1.36	1.91	1.85	2.02	1.66	1.33	1.39	0.56	0.79	0.79	3.45	2.78	2.63	3.75
GER	3.98	4.17	4.05	4.22	4.05	3.88	3.90	3.91	3.82	4.47	3.88	3.92	3.11	3.56	3.33	5.56	3.97	4.31	3.70	2.63	1.25
ITA	7.96	7.32	7.64	7.49	7.62	7.87	7.67	3.40	3.50	2.93	2.95	3.02	2.96	3.25	5.00	6.35	3.97	4.31	4.63	4.39	3.75
GBR	1.57	1.58	1.74	1.64	1.55	1.53	1.76	0.85	1.27	0.92	1.24	1.06	0.89	0.77	3.33	3.17	0.79	1.72	0.93	1.75	0.63
IRL	0.60	0.68	0.81	0.82	0.83	0.59	0.75	0.85	0.80	1.08	0.78	0.60	0.59	0.77	1.11	0.00	0.00	0.00	0.00	0.00	0.63
Rest—Eastern	3.38	3.27	3.47	3.40	3.57	3.17	3.40	3.06	3.18	2.47	2.17	1.66	1.92	2.32	3.33	6.35	7.14	7.76	5.56	4.39	3.13
Rest—Western	10.49	10.25	10.19	10.07	9.88	10.22	10.57	5.61	5.73	6.16	6.51	6.94	6.95	6.97	8.33	7.14	7.14	6.03	8.33	8.77	8.13
**Sub-total**	**31.48**	**30.52**	**31.02**	**30.44**	**30.12**	**30.20**	**30.57**	**19.05**	**20.22**	**19.88**	**19.53**	**18.85**	**17.75**	**19.04**	**25.00**	**29.37**	**23.81**	**27.59**	**25.93**	**24.56**	**21.25**
**Asia -Rest World**																					
IND	25.09	25.56	25.35	25.18	26.19	27.14	27.92	20.07	20.06	21.26	20.93	21.42	21.15	22.76	23.33	20.63	24.60	25.86	29.63	26.32	26.88
CHN	17.85	18.02	18.63	19.32	19.05	17.51	16.86	4.76	5.57	5.39	6.67	6.94	6.95	7.59	10.56	11.90	13.49	14.66	12.96	14.91	15.00
ISR	0.72	0.90	0.93	0.94	0.95	0.82	0.88	1.02	0.80	0.92	0.93	0.90	1.04	0.93	1.11	0.79	0.79	0.86	0.93	0.88	0.63
TWN	2.05	1.80	1.74	1.76	1.67	1.65	1.76	1.70	1.91	2.16	2.02	2.26	2.51	2.48	1.67	3.17	3.17	1.72	0.93	1.75	2.50
SKR	0.84	0.68	0.81	0.94	0.83	0.94	0.88	0.00	0.00	0.00	0.16	0.00	0.00	0.15	1.11	0.79	0.00	0.86	0.00	0.00	0.00
Rest—World	4.58	4.39	4.75	4.57	4.29	4.47	4.53	2.72	3.03	3.08	2.48	2.41	2.37	2.48	5.00	7.14	7.94	2.59	3.70	4.39	3.13
**Sub-total**	**51.15**	**51.35**	**52.20**	**52.69**	**52.98**	**52.53**	**52.83**	**30.27**	**31.37**	**32.82**	**33.18**	**33.94**	**34.02**	**36.38**	**42.78**	**44.44**	**50.00**	**46.55**	**48.15**	**48.25**	**48.13**
**Share of US by type**	**13.75**	**14.08**	**13.08**	**12.88**	**12.62**	**12.93**	**12.96**	**44.05**	**42.36**	**41.14**	**41.24**	**41.33**	**42.60**	**40.09**	**25.56**	**23.02**	**23.02**	**21.55**	**22.22**	**23.68**	**27.50**
**Share of Ex-US by type**	**86.25**	**85.92**	**86.92**	**87.12**	**87.38**	**87.07**	**87.04**	**55.95**	**57.64**	**58.86**	**58.76**	**58.67**	**57.40**	**59.91**	**74.44**	**76.98**	**76.98**	**78.45**	**77.78**	**76.32**	**72.50**
**Share of US by type of**																					
**All Global sites**	**7.14**	**7.61**	**6.89**	**6.81**	**6.58**	**6.70**	**6.43**	**16.22**	**16.20**	**16.29**	**16.47**	**17.01**	**17.55**	**16.18**	**2.88**	**1.77**	**1.77**	**1.55**	**1.49**	**1.65**	**2.75**
**Share of Ex-US by type**																					
**of All Global sites**	**44.77**	**46.47**	**45.82**	**46.07**	**45.56**	**45.16**	**43.22**	**20.60**	**22.05**	**23.31**	**23.47**	**24.15**	**23.64**	**24.17**	**8.39**	**5.91**	**5.92**	**5.63**	**5.21**	**5.30**	**7.25**
**Share total Global facility types Across all Global sites**	**51.91**	**54.08**	**52.72**	**52.88**	**52.14**	**51.86**	**49.66**	**36.82**	**38.25**	**39.60**	**39.94**	**41.15**	**41.19**	**40.35**	**11.27**	**7.67**	**7.69**	**7.18**	**6.70**	**6.95**	**9.99**

We first compare total counts of manufacturing facility sites based on voluntary self-identification data procurement efforts (data used in the previous research) with counts based on actual payments of mandated GDUFA fees (the new FDA data) over the 2013–2017 overlapping years. As seen in the cells in the bottom right corner of Table 1, between 2013 and 2017 the global total number of API, FDF, and combined API and FDF sites ranged between 1597 and 1642; the 2013–2017 average number is 1621. In our previous research, the 2013–2017 average was 1555.^62^ Although the time trends are quite similar, on average the total number of sites drawn from a list based on actual payment of mandated GDUFA fees (the new data source) is about 4.2 percent larger than on the list relying on FDA’s voluntary self-identification programs (the previous data source). If one separates this into its US and ex-US components, one finds that the average total number of US sites 2013–2017 based on the new data is 411, while at 404 that based on the old voluntary data is 1.7 percent smaller. For ex-US sites, the 2013–2017 total number of sites based on the new data is 1210, while that based on the earlier voluntary data is 5.0 percent smaller at 1152.^63^ Hence the extent of underreporting of manufacturing sites in the old voluntary data compared to the new mandatory data is not quite three times larger for ex-US than for US sites.

### IV.B. Prevalence of Combined API and FDF Sites

As seen in the bottom corner of Table 2, as a share of all global sites, combined sites comprise only from 6.70 to 11.27 percent. Thus, combined sites are not as prevalent as dedicated API or dedicated FDF sites. Combined sites are found more frequently ex-US than in the US; for the US between 2013 and 2019 this share ranges from 1.49 to 2.88 percent and for ex-US sites this share ranges from 5.21 to 8.39 percent. At all three levels of geographic aggregation (US, ex-US and global), a sharp decline in the number of combined sites occurred in 2014, a more gradual decline took place between 2014 and 2018, and then a substantial increase occurred in 2019 (cells in bottom right corner of Table 1). The corresponding variation in shares is shown in the lower right corner of Table 2.

One possibility is that these relatively large changes involving counts of combined API and FDF sites 2013–2014 and 2018–2019 involved firms’ reactions to changed GDUFA-I and GDUFA-II annual user fee assessments depending on whether the site was dedicated API, dedicated FDF, or a combined site.^64^ Under GDUFA-I, the large FDF annual user fee could be avoided by switching from a combined to dedicated API facility, and under GDUFA-II, the penalty for converting from a dedicated API to combined API and FDF facility was much smaller than under GDUFA-I. These facility site change patterns merit further scrutiny.

### IV.C. Counts of Global, US and Ex-US Dedicated and Combined Sites

One set of striking findings in our previous research was that between 2013 and 2017, the global number of API sites was falling. Simultaneously, the US share of global API sites was small and shrinking and the ex-US share was large and increasing. With the new FDA data, the API trend is not as uniform. As seen in the bottom rows of Table 1, while the total number of API sites in the USA fell in all years between 2013 and 2017, a slight increase occurred in 2018 and then a substantial decrease took place in 2019, so that the 2019 number was about 10 percent less than in 2013 (103 vs. 114 sites). In contrast, the number of ex-US API sites both increased and decreased between adjacent years 2013–2018, but then dropped sharply in 2019, with the 2019 number being 3.2 percent less than in 2013. As seen near the bottom panel of Table 2, the US share of global API sites was 13.8 percent in 2013, hovered at between about 12–14 between 2014 and 2018, and ended up at 13 percent in 2019. Thus, the trends using the new FDA data are slightly less monotonic but generally confirm levels and trends of API findings reported in our previous research. API manufacturing is overwhelmingly and increasingly occurring abroad.

Similar calculations involving data reported in the bottom rows of the middle panel in Table 2 reveal that the US share of global FDF sites fell from 44.0 to 40.1 percent between 2013 and 2019, although the decline is not monotonic. The extent to which ex-US shares dominate US shares is smaller but increasing for FDF sites. Why the USA has a larger share of FDF than API sites may reflect that fact that the shipping of commodity API materials may be more robust, less fragile and less costly than that for more aseptic, temperature and light-sensitive finished FDF products requiring greater manufacturing and regulatory sterile quality oversight. These patterns merit further scrutiny. Finally, the combined sites are predominantly ex-US, as seen in the lower right corner of Table 2; the US share is 25.6 percent in 2013, ranges between 21.6 and 23.7 percent in 2014–2018, and then increases to 27.5 percent in 2019.

To summarize, facilities manufacturing generic prescription pharmaceuticals destined for US consumption are all increasingly located in ex-US geographies; ex-US locations are more prevalent for API than for FDF facilities. These findings are generally consistent with those previously published using less detailed data.

### IV.D. Facility Counts by Detailed Geographic Location

We now move to a discussion of findings based on the more disaggregated global data reported in Tables 1 and 2 calculated using the new data. We begin with API site locations (columns in left panel) then discuss FDF site locations (columns in middle panel), and finally report on the disaggregated locations of simultaneous API and FDF sites (columns in right panel).

USA’ dependence on imported API is large, and is increasingly largely sourced from India and China. Over the entire 2013–2019 time period, both India and China hosted more API sites than the USA (respective averages of about 220, 154, and 112), with Italy (average about 65) and Germany (average about 34) rounding out the top five countries by API site count. In terms of trends, the number of API sites in India increased in 2014, and has stabilized since then. In China, the number of API sites increased in 2014, stabilized 2014–2017, then fell in 2018 and fell even more so in 2019 so that by 2019 the number of API sites in China was not quite 10 percent less than in 2013. When summed over India and China API sites, the combined share of global API sites was 42.9 percent in 2013, 44.5 percent in 2016, and 44.8 percent in 2019. The number of API sites in Italy declined from 66 in 2013 to 61 in 2019, and for Germany from 33 to 31.

The USA, Canada and E.U. member countries play a more prominent role among FDF than API sites. Since 2014, India (2014–2019 average about 138) and China (average about 42) have hosted much fewer FDF sites than the USA (average 270). Among FDF sites, Canada (average about 29) and Germany (average about 25) rounded up the top five FDF facility counts; Italy (average about 20) is in sixth place. Although for many countries there is a decline in 2019 in the number of FDF sites, over the entire 2013 time period the number of FDF sites decreased in Canada, and stabilized in Germany and Italy. While the number of FDF sites in India is greater than in China, the growth is more rapid in China. The share of global FDF sites summed over India and China is growing more rapidly than for API sites. Whatever advantages the USA had historically in hosting FDF manufacturing facilities, those advantages appear to be decreasing.

Finally, combined sites are largely ex-US sourced. Among combined sites over the 2013–2019 time frame, seen in the columns on the right panel of Table 1, India hosted the most sites (average about 33), followed closely by the USA (average about 32), and more distantly by China (average about 18), while no other country averaged over 10 sites, with Italy (average about 6) and Germany (average about 5) rounding out the top five countries. When summed over India and China, the share of global combined sites was 33.9 percent in 2013, 40.5 percent in 2016, and 41.9 percent in 2019.

### IV.E. Facility Counts by US Geographic Location

In Table 3, we present annual 2013–2019 levels of API (left panel), FDF (middle panel), and combined (right panel) site counts for the USA, within the USA for the 19 FDA districts, and for the US districts aggregated into four zones *(Southeast, Other East, Central* and *West),* as defined in Section IV.B. above. In Table 4, we present corresponding shares of district and zone site counts in the USA.

**Table 3:** Number of dedicated API, dedicated FDF, and simultaneous API and FDF sites by FDA region, FY 2013–2019

	**Number of dedicated API sites**							**Number of dedicated FDF sites**							**Number of simultaneous API and FDF sites**						
**FDA regions**	**2013**	**2014**	**2015**	**2016**	**2017**	**2018**	**2019**	**2013**	**2014**	**2015**	**2016**	**2017**	**2018**	**2019**	**2013**	**2014**	**2015**	**2016**	**2017**	**2018**	**2019**
**Southeast**																					
ATL	14	15	15	13	14	15	14	25	27	27	31	27	34	30	5	4	3	1	1	1	1
FLA	0	0	0	1	1	1	1	18	17	18	16	16	17	8	1	0	0	1	1	0	2
NOL	7	9	9	7	7	7	5	7	6	4	8	7	7	5	4	4	2	1	1	0	1
SJN	6	5	4	3	2	2	3	6	8	9	11	10	9	9	3	1	2	1	2	1	2
**Sub-total**	**27**	**29**	**28**	**24**	**24**	**25**	**23**	**56**	**58**	**58**	**66**	**60**	**67**	**52**	**13**	**9**	**7**	**4**	**5**	**2**	**6**
**Other East**																					
BLT	2	2	2	1	1	1	1	7	9	10	9	9	9	9	1	0	0	0	0	0	0
NWE	6	6	4	4	4	4	4	11	10	10	10	11	11	9	1	0	0	0	0	1	0
NWJ	9	12	12	12	10	10	8	38	40	38	39	45	44	40	6	4	7	4	4	4	8
NYK	5	6	5	5	5	7	6	34	35	33	30	32	36	34	2	0	0	0	0	1	6
PHI	6	10	9	7	9	10	8	17	17	17	17	18	17	14	5	3	4	3	3	4	6
**Sub-total**	**28**	**36**	**32**	**29**	**29**	**32**	**27**	**107**	**111**	**108**	**105**	**115**	**117**	**106**	**15**	**7**	**11**	**7**	**7**	**10**	**20**
**Central**																					
CHI	8	12	9	9	10	10	9	14	12	13	11	10	11	11	3	2	1	2	2	2	4
CIN	9	10	9	8	7	8	7	12	12	13	12	12	13	14	3	2	2	2	2	2	3
DAL	6	6	5	5	4	4	3	11	11	11	10	11	10	9	2	1	1	1	1	3	1
DET	7	5	3	5	5	5	5	12	16	15	14	14	14	14	5	2	3	3	1	1	2
KAN	13	10	10	10	10	10	12	8	7	8	8	8	9	9	1	2	1	2	2	4	4
MIN	8	7	6	5	6	5	6	5	6	6	7	6	8	7	0	0	1	1	1	1	1
**Sub-total**	**51**	**50**	**42**	**42**	**42**	**42**	**42**	**62**	**64**	**66**	**62**	**61**	**65**	**64**	**14**	**9**	**9**	**11**	**9**	**13**	**15**
**West**																					
DEN	3	4	4	6	5	4	4	11	10	12	12	12	12	12	1	2	1	2	2	1	2
LOS	5	5	6	7	5	7	6	19	20	19	17	22	21	21	3	1	1	1	1	1	1
SAN	0	0	1	2	1	0	1	4	3	3	3	3	4	3	0	1	0	0	0	0	0
SEA	0	1	0	0	0	0	0	0	0	1	1	1	1	1	0	0	0	0	0	0	0
**Sub-total**	**8**	**10**	**11**	**15**	**11**	**11**	**11**	**34**	**33**	**35**	**33**	**38**	**38**	**37**	**4**	**4**	**2**	**3**	**3**	**2**	**3**
**Total US by type**	**114**	**125**	**113**	**110**	**106**	**110**	**103**	**259**	**266**	**267**	**266**	**274**	**287**	**259**	**46**	**29**	**29**	**25**	**24**	**27**	**44**
**Total US Sites across all types**	**419**	**420**	**409**	**401**	**404**	**424**	**406**	**419**	**420**	**409**	**401**	**404**	**424**	**406**	**419**	**420**	**409**	**401**	**404**	**424**	**406**

**Table 4:** Share of dedicated API, dedicated FDF, and simultaneous API and FDF sites by FDA region, FY 2013–2019

	**Share of dedicated API sites**							**Share of dedicated FDF sites**							**Share of simultaneous API and FDF sites**						
**FDA regions**	**2013**	**2014**	**2015**	**2016**	**2017**	**2018**	**2019**	**2013**	**2014**	**2015**	**2016**	**2017**	**2018**	**2019**	**2013**	**2014**	**2015**	**2016**	**2017**	**2018**	**2019**
**Southeast**																					
ATL	12.28	12.00	13.27	11.82	13.21	13.64	13.59	9.65	10.15	10.11	11.65	9.85	11.85	11.58	10.87	13.79	10.34	4.00	4.17	3.70	2.27
FLA	0.00	0.00	0.00	0.91	0.94	0.91	0.97	6.95	6.39	6.74	6.02	5.84	5.92	3.09	2.17	0.00	0.00	4.00	4.17	0.00	4.55
NOL	6.14	7.20	7.96	6.36	6.60	6.36	4.85	2.70	2.26	1.50	3.01	2.55	2.44	1.93	8.70	13.79	6.90	4.00	4.17	0.00	2.27
SJN	5.26	4.00	3.54	2.73	1.89	1.82	2.91	2.32	3.01	3.37	4.14	3.65	3.14	3.47	6.52	3.45	6.90	4.00	8.33	3.70	4.55
**Sub-total**	**23.68**	**23.20**	**24.78**	**21.82**	**22.64**	**22.73**	**22.33**	**21.62**	**21.80**	**21.72**	**24.81**	**21.90**	**23.34**	**20.08**	**28.26**	**31.03**	**24.14**	**16.00**	**20.83**	**7.41**	**13.64**
**Other East**																					
BLT	1.75	1.60	1.77	0.91	0.94	0.91	0.97	2.70	3.38	3.75	3.38	3.28	3.14	3.47	2.17	0.00	0.00	0.00	0.00	0.00	0.00
NWE	5.26	4.80	3.54	3.64	3.77	3.64	3.88	4.25	3.76	3.75	3.76	4.01	3.83	3.47	2.17	0.00	0.00	0.00	0.00	3.70	0.00
NWJ	7.89	9.60	10.62	10.91	9.43	9.09	7.77	14.67	15.04	14.23	14.66	16.42	15.33	15.44	13.04	13.79	24.14	16.00	16.67	14.81	18.18
NYK	4.39	4.80	4.42	4.55	4.72	6.36	5.83	13.13	13.16	12.36	11.28	11.68	12.54	13.13	4.35	0.00	0.00	0.00	0.00	3.70	13.64
PHI	5.26	8.00	7.96	6.36	8.49	9.09	7.77	6.56	6.39	6.37	6.39	6.57	5.92	5.41	10.87	10.34	13.79	12.00	12.50	14.81	13.64
**Sub-total**	**24.56**	**28.80**	**28.32**	**26.36**	**27.36**	**29.09**	**26.21**	**41.31**	**41.73**	**40.45**	**39.47**	**41.97**	**40.77**	**40.93**	**32.61**	**24.14**	**37.93**	**28.00**	**29.17**	**37.04**	**45.45**
**Central**																					
CHI	7.02	9.60	7.96	8.18	9.43	9.09	8.74	5.41	4.51	4.87	4.14	3.65	3.83	4.25	6.52	6.90	3.45	8.00	8.33	7.41	9.09
CIN	7.89	8.00	7.96	7.27	6.60	7.27	6.80	4.63	4.51	4.87	4.51	4.38	4.53	5.41	6.52	6.90	6.90	8.00	8.33	7.41	6.82
DAL	5.26	4.80	4.42	4.55	3.77	3.64	2.91	4.25	4.14	4.12	3.76	4.01	3.48	3.47	4.35	3.45	3.45	4.00	4.17	11.11	2.27
DET	6.14	4.00	2.65	4.55	4.72	4.55	4.85	4.63	6.02	5.62	5.26	5.11	4.88	5.41	10.87	6.90	10.34	12.00	4.17	3.70	4.55
KAN	11.40	8.00	8.85	9.09	9.43	9.09	11.65	3.09	2.63	3.00	3.01	2.92	3.14	3.47	2.17	6.90	3.45	8.00	8.33	14.81	9.09
MIN	7.02	5.60	5.31	4.55	5.66	4.55	5.83	1.93	2.26	2.25	2.63	2.19	2.79	2.70	0.00	0.00	3.45	4.00	4.17	3.70	2.27
**Sub-total**	**44.74**	**40.00**	**37.17**	**38.18**	**39.62**	**38.18**	**40.78**	**23.94**	**24.06**	**24.72**	**23.31**	**22.26**	**22.65**	**24.71**	**30.43**	**31.03**	**31.03**	**44.00**	**37.50**	**48.15**	**34.09**
**West**																					
DEN	2.63	3.20	3.54	5.45	4.72	3.64	3.88	4.25	3.76	4.49	4.51	4.38	4.18	4.63	2.17	6.90	3.45	8.00	8.33	3.70	4.55
LOS	4.39	4.00	5.31	6.36	4.72	6.36	5.83	7.34	7.52	7.12	6.39	8.03	7.32	8.11	6.52	3.45	3.45	4.00	4.17	3.70	2.27
SAN	0.00	0.00	0.88	1.82	0.94	0.00	0.97	1.54	1.13	1.12	1.13	1.09	1.39	1.16	0.00	3.45	0.00	0.00	0.00	0.00	0.00
SEA	0.00	0.80	0.00	0.00	0.00	0.00	0.00	0.00	0.00	0.37	0.38	0.36	0.35	0.39	0.00	0.00	0.00	0.00	0.00	0.00	0.00
**Sub-total**	**7.02**	**8.00**	**9.73**	**13.64**	**10.38**	**10.00**	**10.68**	**13.13**	**12.41**	**13.11**	**12.41**	**13.87**	**13.24**	**14.29**	**8.70**	**13.79**	**6.90**	**12.00**	**12.50**	**7.41**	**6.82**
**US Share of Global by type**	**13.75**	**14.08**	**13.08**	**12.88**	**12.62**	**12.93**	**12.96**	**44.05**	**42.36**	**41.14**	**41.24**	**41.33**	**42.60**	**40.09**	**25.56**	**23.02**	**23.02**	**21.55**	**22.22**	**23.68**	**27.50**

As discussed earlier, in the entire USA there are about twice as many FDF as API sites, with the number of combined sites being a distant third. For API manufacturing, it is the *Central* zone that dominates, while for FDF and combined API and FDF manufacturing it is the *Other East* zone that dominates. In general, the greatest pharmaceutical manufacturing concentration within the US geography is for FDF manufacturing in the *Other East* zone consisting of New Jersey, New York, Philadelphia, New England, and Baltimore FDA districts. Notably, these FDF locations are each within easy one-day travel distance from FDA headquarters in suburban Washington DC, facilitating exploitation of agglomeration economies. API sites are distributed more evenly among the four zones than FDF sites (consistent with API transport costs being largely irrelevant), although API manufacturing is the one facility type in which the “rust belt” *Central* zone dominates. FDF manufacturing is the most prevalent facility type for the *Southeast* zone, although FDF manufacturing is most prevalent in the *Other East* zone. The *Central* and Other East zones each have on average about 11 combined sites, much greater than in the *Southeast* (average not quite seven) or *West (*average of three) zones. There is some vacillation in the top three zone rankings among *Central, Other East* and *Southeast* zones, depending on the API, FDF, or combined facility type, but uniformly the *West* zone has the fewest manufacturing facilities across all site types.

In terms of changes rather than levels, although over the entire USA the number of API sites fell 2013–2019, the *West* zone bucked the national trend, with the number of API sites there increasing from 8 to 11. The *Central* zone bore the largest brunt of the national API decline, with the number falling by almost 20 percent from 51 to 42; the Dallas district lost three sites, the Minnesota and Detroit districts each lost one site, even as Cincinnati gained a site. The number of API sites in the *Other East* zone was relatively stable at 27–28, while for the *Southeast* zone, it fell from 27 to 23, with San Juan losing three sites (from six to three) and New Orleans two (from seven to five).

When summed across all three facility types (API, FDF, and combined), over the 2013–2019 time period the *Southeast* zone is losing the most manufacturing sites (from 96 to 81), more than the *Central* zone (127 to 121) but there are slight gains in the *Other East* zone (150 to 153) and proportionally larger ones in the *West* (from 46 to 51). Notably, when compared to the size of facility shift counts globally—particularly involving India and China—within the USA shifts in geographic location of generic drug manufacturing are relatively modest. The global shift from the USA to India and China is being felt within the USA primarily by manufacturing site count declines in the *Southeast* and *Central* zones, but even within the *Southeast* zone there are FDA districts experiencing both increases and decreases in the number of manufacturing facilities.

## V. SUMMARY OF FINDINGS AND IMPLICATIONS

The goal of this research has been to document the changing geography of generic drug manufacturing between 2013 and 2019 among those pharmaceuticals intended to be consumed in the USA.

What we have found is that even in an era where the number of generic drug prescriptions dispensed in the USA grew by about 15 percent,^65^ the global total number of API sites fell by about 5 percent and the global total of FDF sites increased by just over 5 percent. From an economic perspective, we interpret the larger proportionate reduction in API than FDF facilities, both domestic and foreign that we document, is likely the result of firms’ attempting to capture scale economies by increasing plant capacity, and for US firms, exploiting comparative advantages by outsourcing generic drug manufacturing to foreign FDF, and especially API suppliers.

API intended for the US market is overwhelmingly manufactured ex-US. The ex-US share has been relatively stable at about 87 percent, with the ex-US API sources dominated by India (about 26 percent of global) and China (18 percent). When summed over India and China, their combined share increased from about 42 percent to 45 percent. Meanwhile, API shares for the fourth and fifth ranked countries—Italy and Germany—fell slightly to about 8 percent and 4 percent, respectively.

In terms of FDF production, unlike for API, the USA is still the largest supply source in terms of site counts (averaging about 41 percent of global sites), India is second (growing to about 21 percent), while China is third (with share growing from 5 percent to 8 percent). As the share of global FDF sites has increased in India and China, it fell slightly in the USA, but was stable in Europe where Germany and Italy have a combined share of about 6 percent. Why the ex-US share of FDF sites lags the ex-US share of API merits further attention; it is possible that the availability of scale economies is geographically neutral facilitating ubiquitous API manufacturing, but that final consumption geographic preferences depend more on agglomeration economies and favor siting FDF facilities near the location of final drug manufacturing, oversight, and consumption. This interpretation merits additional research.

Within the USA, the total number of API sites declined by about 10 percent between 2013 and 2019, with the decline borne entirely by two FDA regions—the *Central* zone that lost 10 sites (four in DAL and three in DET) and the *Southeast* zone that lost nine (with five being in SJN and four in NOL). Thus, API “losers” in the USA were regions vulnerable to supply interruptions from severe weather and the “Rust Belt” states.

Although the total number of FDF sites in the USA was unchanged between 2013 and 2019, the *Southeast* zone lost the most share, particularly FLA and NOL, even as the Carolinas and Georgia experienced a slight increase, and SJN vacillated, growing in 2013–2016, but then falling to 2019 after Hurricane Maria.

Based on these results that confirm previous studies, it should come as no surprise that the US pharmaceutical supply chain is susceptible to the outcomes of recent and ongoing international trade negotiations, to climate and weather pattern calamities, other natural disasters, exposure to disease pandemics such as COVID-19, and engagement in military conflicts. Because of the USA’ substantial reliance on imported API and FDF from India and China, current bilateral international trade negotiations with either of them and between them could have a material impact on domestic prices of generic drugs.^66^ By comparison, the very limited imports from the UK, Ireland, Mexico, and Canada suggest that the Brexit and USA–Mexico–Canada trade negotiations will have at most modest impacts on domestic generic drug prices.^67^ Military engagements could also impact domestic availability of generic drugs, but currently few Russian and Mideastern sites import API and FDF into the USA, although USA’ dependence on Chinese manufacturing of antibiotics is substantial.^68^ Global climate change patterns and severe weather events such as flooding could potentially affect pharmaceutical supplies. Within the USA we find there has been a substantial reduction in the number of API and FDF sites in severe weather vulnerable regions such as the *Southeast* and *Central* zones.

The implications of our research regarding the USA’ dependence and potential vulnerability to foreign pharmaceutical supplies highlights a significant data limitation of this research, a limitation that has also been exposed as FDA has sought to deal with drug shortages over the last decade: We simply do not know, as one researcher stated, “Who Makes This Drug?”^69^ Because of the increasingly common industry practice of outsourcing manufacturing to contract manufacturing organizations and the failure of firms promptly to notify FDA or the public of product quality issues and product discontinuation, according to this researcher,

“Even FDA can’t easily determine whether a drug is made by the ANDA sponsor or a contract manufacturer … FDA maintains records that identify which manufacturers are producing generic drugs for the US market. However, these data aren’t maintained in a format that makes it possible for the agency to quickly distinguish between ANDA holders and contract manufacturers of fill and finish products or base ingredients. These records aren’t available for public scrutiny … .The agency generally treats nonpublic business relationships as confidential commercial or financial information, exempting it from public disclosure.”^70^

Although the API and FDF manufacturing site data made available to us by FDA identify the address of the site and the organization paying the Generic Drug User Fee, no information is available in these data identifying the products actually manufactured at the site nor their volumes. As FDA’s Drug Shortage Task Force simply stated, “FDA’s data do not capture how much of a drug is produced at each manufacturing facility.” ^71^ Even FDA data revealing results of FDA manufacturing site inspections “does not list the products being made at the facilities”.^72^ According to FDA, “… the pharmaceutical industry regards the location of their manufacturing facilities as confidential commercial information and claims that keeping this information private is a matter of supply security, eg to prevent theft or diversion attempts.”^73^

Moreover, many generic drugs whose ANDA has been FDA approved are not marketed. For example, as FDA acknowledged in its Task Force study of Drug Shortages,

“FDA analysis shows that as of June 2019, for all generic drugs with approved applications, 39 percent were observed to be marketed, and the remaining 61 percent were approved but not marketed.”^74^

The phenomenon of FDA-approved generic drugs not actually being marketed is occurring even for FDA’s prioritized “first generics”—those drugs approved by FDA as the first competitor to a brand that has lost marketing exclusivity, and considered by FDA to be a public health priority bringing new competition and savings to the US market. A 2019 study published by the Association for Accessible Medicines—the USA’ generic drug trade association—concluded that “… fewer than half of the first generics approved by FDA since 2016 are commercially available to patients”.^75^

Other data-imposed limitations of this research include the fact that our analysis is restricted to generic small molecule prescription drug manufacturing. Whether the manufacturing dichotomy between API and FDF sites is as relevant for biologics and biosimilars as it is for small molecules is unknown. More generally, research on what the evolution of the geography of generic drug manufacturing teaches us regarding how the economic geography of biosimilar manufacturing, distribution, and regulation will evolve would be most useful. Finally, in terms of policy, in the wake of the COVID19 pandemic, reports of shortages, alleged price gouging, and the “hoarding” of certain medical products, including some generic prescription drugs, have emerged as concerns among American hospitals, state governors, and the Centers for Disease Control and Prevention. These challenges have highlighted the results of this and other research to a wide range of stakeholders that Americans depend on prescription drugs that are largely manufactured by global companies operating in Southeast Asia and Europe. They also suggest that American dependency on generic drug supply from ex-US sources may be amenable to additional government policies and regulations.

In facing the incident crisis, the Trump Administration has made clear that local hospitals, individual cities, and the states should be self-sufficient in sourcing products, forcing them to compete against each other as well as the federal government and other countries for FDF products and for firms competing for access to API materials. With each American hospital sourcing these products alone, hospital procurement officers have run into a bewildering array of middlemen, international procurement rules, import quotas and allegedly exorbitant freight charges.^76^ Supply quality is also emerging as a concern as the USA transitions to managing COVID19 as a chronic public health crisis. Hospitals and states have reported encountering unvetted brokers and counterfeit goods. Moreover, the finances of hospitals, particularly those that serve the safety net, are now emerging as at risk.^77^ For some hospitals and communities, the purchasing of needed drugs at exorbitant ‘crisis’ prices might become an existential threat.^78^

It is useful to note that dependency on ex-US sources of supply is not a new problem, and is not unique to the pharmaceutical industry. Non-pharmaceutical firms deal with supply vulnerabilities on a daily basis as part of their routine risk management operations. Whether the tools they employ are adequate to protect our nation’s pharmaceutical supply chain merits close inspection, but it is clear to us that it would be unwise for prudent industry and public policy involving routine risk management to be replaced by actions motivated by xenophobia concerns. Nonetheless, the outcomes of private sector risk management of supply chains may differ from those informed by global political considerations and pandemics, and scrutiny of whether externalities from private sector actions need to be evaluated.

American policymakers appear to be interested in pursuing policies that would reduce considerably America’s traditional reliance on the global supply chain. These include rules and preferences mitigating reliance on a few countries that currently generate the bulk of the basic ingredients and finished medical products, most notably China and India. As we were writing this piece, President Trump signed the “Buy American” Executive Order.^79^ The Order outlines an initiative to review all federal purchases with preference to buy American-made goods. It also includes additional directives to federal agencies to recommend ways to strengthen the implementation of ‘Buy American’ laws including domestic procurement preference policies and programs.

Here, additional policies might be considered to encourage production of raw materials for crucial medical supplies and raw ingredients for pharmaceuticals and other needed medical products that would be available in the USA in the event of international disruptions. Policies might include favorable economic incentives and environmental regulations for US companies to ensure that material is locally sourced, stored and that manufacturing facilities can be adapted for rapid production. Federal price gouging rules might also be considered. Whether pursuing a national policy of “generic drug independence” makes any more sense than our previous national policy of achieving “US energy independence”, and whether generic drug independence is both wise and feasible, deserves wide discussion and consideration.

The benefits of these and other efforts need to be carefully considered, as in practice it will take time to increase domestic manufacturing capabilities. There are also costs to consider in pursuing these policies. Diversification of medical supply production to the USA and other higher income countries will increase costs of goods, inflicting some additional burden on our health care system.

In the wake of the COVID19 crisis, FDA also activated numerous pathways to assure the reliability and quality of prescription drugs. On the other hand, the FDA also suspended inspections of many existing products that the agency regulates,^80^ likely contributing to delays in increasing the supply of these products and putting the quality of these products over time at risk.

In summary, while we believe this research contributes substantively to our understanding of where in the globe generic drug API and FDF manufacturing facilities are located and how that is changing, a major gap in our knowledge meriting high research priority awaits data availability linking site locations to products actually being manufactured, and their volumes. We leave it to future empirical work to further empirically investigate the associative and causal relationships between country-specific policies and drug manufacturing location. Another illuminating empirical exercise would be to undertake a similar descriptive examination of drug manufacturing among products destined for European Union consumption. The COVID19 pandemic has underscored the importance of this and future research on American’s reliance on the global supply of generic prescription drugs.

## Footnotes

1
https://www.consumerreports.org/prescription-drugs/too-many-meds-americas-love-affair-with-prescription-medication/

2 IQVIA Institute for Human Data Science, *Medicine Use and Spending in the U.S.: A Review of 2017 and Outlook to 2022,* Parsippany, NJ, April 2018, p, 14. Available online https://www.iqvia.com/institute/reports....

3
https://www.cbsnews.com/news/worse-than-you-think-10-things-you-dont-know-about-glaxos-750m-paxil-settlement/

4 For one account focusing on drug quality and safety controversies, see Katherine Eban, *Bottle of Lies: The Inside Story of the Generic Drug Boom*, New York: HarperCollins Publishers, 2019. Concerns over the US Food and Drug Administration’s track record in inspecting foreign manufacturing sites led the US Congress in 2012 in part to pass the Generic Drug User Fee Act legislation. For discussion, see Ernst R. Berndt, Rena M. Conti and Stephen J. Murphy, “The generic drug user fee amendments: an economic perspective”, *Journal of Law and the Biosciences*, lsy002, https:doi.org/10.1093/jlb/lsy002, published 11 April 2018. For a discussion of drug shortage issues, see Christopher Stomberg, “Drug Shortages, Pricing and Regulatory Activity”, ch. 10 in Ana Aizcorbe, Colin Baker, Ernst R. Berndt and David M. Cutler, eds., *Measuring and Modeling Health Care Costs*, Chicago: University of Chicago Press for the National Bureau of Economic Research, 2018, pp. 323–348; and Rena M. Conti and Ernst R. Berndt, “Specialty Drug Prices and Utilization After Loss of US Patent Exclusivity, 2001–2007”, ch. 9 in Aizcorbe, Baker, Berndt and Cutler [2018], *op. cit.*, pp. 273–321.

5 Rena M. Conti and Ernst R. Berndt, “Four Facts Concerning Competition in U.S. Generic Prescription Drug Markets”, *International Journal of the Economics of Business*, August 2019. DOI: 10.1080/13571516.2019.1654324.

6 Ernst R. Berndt, Rena M. Conti and Stephen J. Murphy [2018], *op. cit.* Jasdeep Singh, Mohit Jain and Pratyush Goel, *US Generics Market—Evolution of Indian Players*, IQVIA Institute for Human Data Science, February 2019. Available online from www.IQVIA.com; Kyle Blankenship, “As API production consolidates abroad, U.S. regulators face safety concerns at home”, *Fierce Pharma*, August 29, 2019. Available online https://www.fiercepharma.com/pharma/half-fda-warnings-letters-last-year-were-seng-to-in…

7 For one account focusing on drug quality and safety controversies, see Katherine Eban [2019], *op. cit.* Concerns over the US Food and Drug Administration’s track record in inspecting foreign manufacturing sites led the US Congress in 2012 in part to pass the Generic Drug User Fee Act legislation. For discussion, see Ernst R. Berndt, Rena M. Conti and Stephen J. Murphy [2018], *op. cit.* For a discussion of drug shortage issues, see Christopher Stomberg [2018], *op. cit.;* and Rena M. Conti and Ernst R. Berndt, [2018], *op. cit.*,

8 Conti RM, Nguyen KH, Rosenthal MB. “Generic prescription drug price increases: which products will be affected by proposed anti-gouging legislation?” *J Pharm Policy Pract*. 2018 Nov 21;11:29.

9 Scott R. Atlas, W.H. McMaster. “Relying on Foreign Drugs is Dangerous: Generics are often made in India, with ingredients from China. Time to diversify the supply chain.” *Wall Street Journal*, 2020 April 28. Available online: https://www.wsj.com/articles/relying-on-foreign-drugs-is-dangerous-11588093635

10
https://www.fda.gov/drugs/drug-information-consumers/protecting-americas-health-through-human-drugs

11 Berndt, Conti and Murphy [2018], *op. cit.*

12
https://www.registrarcorp.com/fda-drugs/definitions/.

13 The US Food and Drug Administration defines active pharmaceutical ingredient as “Any substance or mixture of substances intended to be used in the manufacture of a drug (medicinal) product and that, when used in the production of a drug, becomes an active ingredient of the drug product. Such substances are intended to furnish pharmacological activity or other direct effect in the diagnosis, cure, mitigation, treatment, or prevention of disease or to affect the structure or function of the body”. See US FDA Drug Definitions, available online: https://www.registrarcorp.com/fda-drugs/definitions/.

14 See https://www.fda.gov/media/73549/download

15 FDA explains the rationale for its central focus on protecting consumers from drugs that do not meet these standards on its webpage, titled “Promoting safe and effective drugs for 100 years’, as follows: “At the turn of the 20th century there were no federal regulations to protect the public from dangerous drugs. ‘It was a menacing market.’….’Products …were at minimum, useless remedies that picked the pocket of the user, but they could also be downright harmful.’” See https://www.fda.gov/about-fda/histories-product-regulation/promoting-safe-effective-drugs-100-years

16 FDA’s portion of the Code of Federal Regulations (*CFR)* is in Title 21, which interprets the *Federal Food, Drug and Cosmetic Act* and related statutes, including the Public Health Service Act. The pharmaceutical or drug quality-related regulations appear in several parts of Title 21, including sections in parts 1–99, 200–299, 300–499, 600–799, and 800–1299. 21 CFR Part 314 and Part 600 entails the application and licensing submission requirements for new and generic drug applicants; 21 CFR Part 210 entails current Good Manufacturing Practice in Manufacturing Processing, packing, or Holding of Drugs; 21 CFR Part 211 entails current Good Manufacturing Practice for Finished Pharmaceuticals.

17
https://www.fda.gov/drugs/pharmaceutical-quality-resources/current-good-manufacturing-practice-cgmp-regulations

18 The information discussed in this paragraph and in the next paragraph is taken in large part from a 44-slide Powerpoint presentation by Arthur B. Shaw, Ph.D., FDA DMF Expert, FDA Small Business Office Webinar, Feb. 11, 2013, *Drug Master Files Under GDUFA: DMF Basics,*  https://www.fda.gov/downloads/drugs/developmentapprovalprocess/.../ucm339118.

19 Priyanka Bajpai, “The Changing API Industry”, *BioSpectrum Asia Edition*, 17 December 2018, page 3. Available online: https://www.biospectrumasia.com/opinion/33/12303/the-changing-api-industry.html.

20
*Ibid.,* p. 4.

21 For further discussion, see Ernst R. Berndt, Rena M. Conti and Stephen J. Murphy [2018], *op. cit*.

22
*Ibid*., p. 16.

23 Ernst R. Berndt, Rena M. Conti and Stephen J. Murphy [2018], *op. cit.*

24
*Ibid*., p. 18 of 67.

25 For an extended discussion of the Heckscher-Ohlin framework, see Paul Krugman, Maurice Obstfeld and Marc J. Melitz, *International Trade: Theory and Policy*, Eleventh Edition, New York: Pearson Education, Inc., 2018, especially chapter 5, “Resources and Trade: The Heckscher-Ohlin Model”, pp. 87–122.

26 See, for example, Krugman, Obstfeld and Melitz [2018], *op. cit*., especially chapters 7 (“External Economies of Scale and the International Location of Production”) and 8 (“Export Decisions and Outsourcing”), pp. 151–169 and 170–213, respectively.

27 Alfred Marshall, *Principles of Economics*, 1890. Reprinted London: Macmillan 1920 (8^th^ edition). For a more contemporary discussion, see Michael E. Porter, *The Competitive Advantage of Nations*, New York: The Free Press, 1990, pp. 154–157.Th

28 Edward L. Glaeser, “Introduction”, in Edward L. Glaeser, ed., *Agglomeration Economics*, Chicago: University of Chicago Press for the National Bureau of Economic Research, 2010, p. 1.

29 From basic geometry it is known that the area of a sphere or cylinder (eg boilers or reactors) varies as the two-thirds power of volume, implying that the surface area increases less rapidly than the potential volume, generating product- and possibly plant-specific internal economies of scale. In the engineering literature this is known as the “two-thirds” or “six-tenths” rule, and it implies a rule of thumb that construction costs should increase by only 60–67% as output or capacity output doubles. For empirical support, see, for example, John Haldi and David Whitcomb, “Economies of Scale in Industrial Plants”, *Journal of Political Economy*, August 1967, 75(5):373–385. This intuition is often applied by engineers in estimating the cost of constructing new process equipment, such as that for bulk powders and active pharmaceutical ingredient processes.

30 For example, the Chinese town of Qiaotou, that produces 60% of the world’s buttons and a large proportion of its zippers. The industry’s origins lie in historical accident, but have been amplified by external scale economies. “In 1980, three brothers spotted some discarded buttons in the street, retrieved and sold them, and then realized there Hwas money to be made in the button business…The town’s button and zipper production is carried out by hundreds of small, family-owned firms. Yet there are clearly advantages to each of these small producers in operating in close proximity to the others.” Krugman, Obstfeld and Melitz [2018], *op. cit*., p. 161. While China’s role in the 1980s and 1990s as a huge exporter of labor-intensive products surely reflected in part the forces of comparative advantage, many of these labor-intensive goods were produced by highly localized industries that benefited strongly from external economies of scale.

31 For a discussion of various international trade public policies, see Part 2, “International Trade Policy” in Krugman, Obstfeld and Melitz [2018], *op. cit.,* chapters 9–12.

32 “Puerto Rico’s Pharmaceutical Industry ‘Terminally Ill’,”[2007], November 19, 2007, pp. 2 and 3 o 4. Available online https://www.manufacturing.net/news//2007/11/puerto-ricos-pharmaceutical-industry-terminally... Nonetheless, citing a November 6, 2017 US Food and Drug Administration press release, a 2018 Department of Homeland Security study claimed “According to FDA, Puerto Rico produces 40 billion dollars-worth of pharmaceutical products yearly, more than any other US state or foreign country, by value”. See Department of Homeland Security [2018], *Threats to Pharmaceutical Supply Chains: The Public-Private Analytic Exchange Program, Research Findings*, July 2018, p. 4 of 18. Available online https://www.dhs.gov/sites/default/files/publications/2018_AEP_Threats_to_Pharmaceutical_Supply_Chains.pdf.

33 “The End of Section 936”, *Puerto Rico Report*, posted August 29, 2016. Available online https://www.puertoricoreport.com.

34 “Puerto Rico’s Pharmaceutical Industry ‘Terminally Ill’,” November 19, 2007, ***op. cit***., pp. 2 and 3 o 4. Available online https://www.manufacturing.net/news//2007/11/puerto-ricos-pharmaceutical-industry-terminally...

35
*Ibid.* Also see Scott Greenberg and Gavin Ekins, “Tax Policy Helped Create Puerto Rico’s Fiscal Crisis”, *The Tax Foundation*, June 30, 2015, pp. 1–4 of 5. Available online https://taxfoundation.org/tax-policy-helped-create-puerto-rico-s-fiscal-crisis/.

36 Manuel Madrid, “How the Tax Cut Sacks Puerto Rico”, *American Prospect*, June 28, 2018, p. 4 of 9. Available online https://prospect.org/article/how-tax-cut-sacks-puerto-rico.

37 For a description of the effects of the September 2017 Hurricane Maria on pharmaceutical operations in Puerto Rico, see Department of Homeland Security [2018]*, op. cit.*

38 William Greene, “The Emergence of India’s Pharmaceutical Industry and Implications for the U.S. Generic Drug Market”, Washington DC: U.S. International Trade Commission, Office of Economics Working Paper No. 2007–05-A, May 2007, p. 2 of 36.

39 Chiranjib Neogi, Atsuko Kamiike, Takahiro Sato, “Identification of Factors Behind Performance of Pharmaceutical Industries in India”, Kobe, Japan: Kobe University, Research Institute for Economics and Business Administration, Discussion Paper DP2012–23, September 28, 2012.

40 Gulshan Akhtar, “Indian Pharmaceutical Industry: An Overview”, *IOSR Journal of Humanities and Social Science*, July–August 2013, 13(3):55,63.

41 Neogi, Kamiike, and Sato [2012], *op. cit*., pp. 4–5 of 35.

42 Associated Chambers of Commerce and Industry of India (ASSOCHAM India) and RNCOS Business Consulting Services, Indian API Market Outlook 2022, 2017, Figures 1–1 and 1–2, pages 1 and 2. In volume terms, of its imported API in 2018, India imported 80% from China. Deepak Patel, “Pharma Sector: 80 percent APIs via Chinese imports despite similar making costs”, *Indian Express*, June 19, 2018. Available online https://indianexpress.com/article/business/business-others/pharma-sector-80-per-cent-apis-via-chinese-imports-despite-similar-making-costs-5222951/.

43 Akhtar [2013], *op. cit*.

44 Angus Liu, “‘Old guard’ generics players yield U.S. lead to Indian up-and-comers: analyst”, *Fierce Pharma*, April 18, 2019. Available online https://www.fiercepharma.com/pharma/generics-old-guard-pack-yields-u-s-scripts-lead-to-next-6-led-by-indian-firms-analyst..., pp. 2, 3, and 5 of 5.

45 Haona Li, He Sun, and Frances J. Richmond, “The Historical Evolution of China’s Drug Regulatory System”, Discussion Paper, University of Southern California, International Center for Regulatory Science, August 2014. Available online https://regulatory.usc.edu/files/2014/08/Evolution_China_Reg_system_2014.pdf.

46 Shannon Bennett, “China’s growing presence in the global supply chain”, *Chemistry Today*, January/February 2012, 30(1), p. 1 of 3.

47
*Wikipedia*, “Pharmaceutical industry in China”, last edited on 7 May 2019, pp. 1,3, 4, 6 of 22. Available online https://en.wikipedia.org/w/index.php?title=Pharmaceutical_industry-in-China&oldid=895942969.

48 Bennett [2012], *op. cit*., p. 1 of 3.

49
*Wikipedia* [2019], *op. cit*., pp. 1 and 4 of 22.

50 Kirsty Barnes, “China to play starring role in AstraZeneca API outsourcing”, *InPharmaTechnologi*st, August 5, 2007, pp. 1–2. Available online https://www.in-pharmatechnologist.com/Article/2007/07/05/China-to-play-starring-role-i…

51 Deepak Patel, “Pharma Sector: 80 percent APIs via Chinese imports despite similar making costs”, *Indian Express*, June 19, 2018. Available online https://indianexpress.com/article/business/business-others/pharma-sector-80-per-cent-apis-via-chinese-imports-despite-similar-making-costs-5222951/, p. 6 of 10.

52
*Wikipedia*, “Corporation tax in the Republic of Ireland”, last updated June 17, 2019, p. 12,13, and 25–29 of 55. Available online https://en.wikipedia.org/w/index.php?title=Corporation_tax_in_the_Republic_of_Ireland.

53 Notably, these incentives are augmented by an Irish immigration policy that admits foreign workers much more easily than currently does the US, and likely the UK if in fact the UK carries out its Brexit plans. For discussion, see Shawn Pogatchnik and Heather Long, “Despite Trump’s ire, Ireland expects to avoid any pain from U.S. tax overhaul”, *Washington Post*, December 13, 2017, p. 2 of 5. Available at www.washingtonpost.com/business/economy/trumps-tax-plan-see...

54 Wikipedia [2019], op. cit., pp. 21–25 of 55.

55 Wikipedia [2019], op. cit., p. 1 of 55.

56 Bernard Mallee, “Protecting Innovation in pharma is in Ireland’s interests”. Irish Times, July 27, 2018, p. 1 of 2. Available online https://www.irishtimes.com/opinion/protecting-innovation-in-phaerma-is-in-Ireland-s-interests-1.3577483.

57 For further discussion, see Sections III—VI of Berndt, Conti and Murphy [2018], *op. cit.*

58 We are grateful to Andreas Schick, Randall Lutter and Qiyu Lu from FDA for making this data available to us in Excel files.

59 As published in https://www.fda.gov/about-FDA/contact-ora/ora-district-directors.

60 We are indebted to Dr. Thomas Henry of FDA Office of the Commissioner, Office of Economics and Analysis, Economics Staff, for pointing this out to us. See https://www.fda.gov/media/101064/download for further details.

61 More specifically, a business operation variable in the new FDA data set providing the basis for computing the annual user fee assessment, records whether among categories such as API/FDF analytical testing, API manufacture, FDF manufacture, pack, and clinical bioequivalence or bioavailability, the respondent checked the API manufacture and/or FDF manufacture categories. For our analysis, if only the API manufacture category was checked, we designate that site as dedicated API; if only the FDF manufacture category was checked, we designate that site as dedicated FDF; if both the API manufacture and FDF manufacture business operation categories were checked, we classify that site as a combined or simultaneous API and FDF site.

62 See Table 2 on page 17 of 67 in Berndt, Conti and Murphy [2018], *op. cit.*

63 This involves a comparison of cells in the bottom row of Table 2, page 17 of 67 in Berndt, Conti and Murphy [2018], *op. cit*., with the sum of the 2013–2017 average of dedicated API plus dedicated FDF plus combined API and FDF in the bottom rows of Table 1 in this manuscript.

64 In 2014 under GDUFA-I, the domestic (foreign) API annual program fee was $34,515 ($49,415), while for FDF it was much larger at $220,152 ($235,152). By 2018, however, when GDUFA-II was in effect, both the API and FDF annual program fee decline from their GDUFA-I levels, and the difference between FDF and API annual program fees was smaller. See Table 3, page 22 of 67 and Figure 3, page 37 of 67, in Berndt, Conti and Murphy [2018], *op. cit.*

65 IQVIA Institute for Human Data Science [2018], *op. cit*., pp. 10–14.

66 Subhadip Sircar and Shruti Srivastata, “India Increases Tariffs on U.S. Goods as Trade War Heats Up”, *Bloomberg News*, June 16, 2019. Available online https://www.bloomberg.com/news/articles/2019-06-16/india-imposes-tariffs-on-u-s-goods-as-global-trade-war-heats-up...; Yanzhong Huang, “U.S. Dependence on Pharmaceutical Products from China”, *Council on Foreign Relations Blog Post,* August 14, 2019. Available online https://www.cfr.org/blog/us-dependence--pharmaceutical-products-china. “China’s new drug law may open door for Indian generic medicines: Report”, The Economic Times, August 27, 2019. Available online https://economictimes.indiatimes.com/industry/healthcare/biotech/pharmaceuticals/chinas-new-drug-law-may...

67 Eric Palmer, “PCI Pharma Services takes steps to prepare for Brexit”, *FiercePharma*, August 28, 2019. Available online https://www.fiercepharma.com/manufacturing/pci-pharma-services-takes-steps-to-prepare-...

68 Huang [2019], *op. cit.*

69 Rena M. Conti, “Who Makes This Drug?”, *The Cancer Letter*, 40(1), January 3, 2014. Available from www.cancerletter.com.

70
*Ibid*., pp. 3–4 of 14.

71 U.S. Food and Drug Administration, *Drug Shortages: Root Causes and Potential Solutions—A Report by the Drug Shortages Task Force*, October 2019, p. 118, footnote 19.

72
*Ibid.*, p. 28, footnote 18.

73
*Ibid.*, p. 45

74
*Ibid.*, pp. 31–32. Notably, these non-marketed data “exclude products for which all applications are listed in the discontinued section of Orange Book, as they have effectively left the market and are no longer available.” (footnote 29, p. 32).

75
Access Denied: Why New Generics Are Not Reaching America’s Seniors, Association for Accessible Medicines, 2019.

76 See for example, https://www.flexport.com/blog/a-perfect-storm-how-the-impact-of-covid-19-has-driven-airfreight-to-historic/

77 See for example https://www.fah.org/fah-ee2-uploads/website/documents/COVID-19_Alert-_U.S._Hospital_-_FINAL.pdf

78 See for example https://www.healthcarefinancenews.com/news/us-hospitals-are-suffering-financial-damage-due-covid-19-pandemic-kaufman-hall-finds

79 See https://www.whitehouse.gov/presidential-actions/presidential-executive-order-buy-american-hire-american/

80 See https://www.washingtonpost.com/health/2020/03/10/fda-suspends-most-inspections-foreign-drug-device-food-manufacturers/

